# Prospective Evaluation of Magnetic Resonance Imaging Features of Magnesium-Based Alloy Screw Resorption in Pediatric Fractures

**DOI:** 10.3390/jcm12083016

**Published:** 2023-04-21

**Authors:** Stephan L. Waelti, Simon Wildermuth, Erik P. Willems, Tim Fischer, Tobias J. Dietrich, Sebastian Leschka, Christoph Matissek, Thomas Krebs, Stefan Markart

**Affiliations:** 1 Department of Radiology and Nuclear Medicine, Children’s Hospital of Eastern Switzerland, 9006 St. Gallen, Switzerland; 2 Department of Radiology and Nuclear Medicine, Cantonal Hospital St. Gallen, 9007 St. Gallen, Switzerland; 3 Clinical Trials Unit, Biostatistics, Cantonal Hospital St. Gallen, 9007 St. Gallen, Switzerland; 4 Department of Pediatric Surgery, Children’s Hospital of Eastern Switzerland, 9006 St. Gallen, Switzerland

**Keywords:** absorbable implants, fractures, growth plate, magnesium, osteomyelitis, soft tissue infections, MRI

## Abstract

Background: The resorption of magnesium-based alloy bioabsorbable screws results in the release of hydrogen gas, which can mimic infection and enter the growth plate. The screw itself and the released gas may also affect image quality. Objective: The evaluation of magnetic resonance imaging (MRI) findings during the most active phase of screw resorption is the objective, with particular focus on the growth plate and to assess for the presence of metal-induced artifacts. Material and Methods: In total, 30 prospectively acquired MRIs from 17 pediatric patients with fractures treated with magnesium screws were assessed for the presence and distribution of intraosseous, extraosseous, and intra-articular gas; gas within the growth plate; osteolysis along the screw; joint effusion; bone marrow edema; periosteal reaction; soft tissue edema; and metal-induced artifacts. Results: Gas locules were found in the bone and soft tissues in 100% of the examinations, intra-articular in 40%, and in 37% of unfused growth plates. Osteolysis and the periosteal reaction were present in 87%, bone marrow edema in 100%, soft tissue edema in 100%, and joint effusion in 50% of examinations. Pile-up artifacts were present in 100%, and geometric distortion in 0% of examinations. Fat suppression was not significantly impaired in any examination. Conclusions: Gas and edema in the bone and soft tissues are normal findings during the resorption of magnesium screws and should not be misinterpreted as infection. Gas can also be detected within growth plates. MRI examinations can be performed without metal artifact reduction sequences. Standard fat suppression techniques are not significantly affected.

## 1. Introduction

Fractures are among the most common causes of presentation in pediatric emergency departments [1,2]. Certain pediatric fractures are treated with osteosynthesis. In order not to interfere with bone growth, the osteosynthesis material is removed in a second surgical procedure after the fracture has united [3]. Every surgery and anesthesia are accompanied by their own surgical and anesthetic risks [4,5]. With the use of biodegradable osteosynthesis material, surgery to remove the material becomes obsolete. Biodegradable magnesium-based alloy screws have shown not only complete resorption in animal models, but they even stimulate bone healing [6,7]. In children, they are explored for the treatment of unstable osteochondritis dissecans, for fractures of the tibial tuberosity, the patella, and for certain elbow fractures, e.g., of the medial humeral epicondyle [8,9,10]. In adults, for example, they are used to treat fractures of the medial malleolus, the scaphoid, and the condylar head of the mandible, and for hallux valgus correction [11,12,13,14,15,16,17,18,19,20].

A characteristic feature of magnesium-based alloy screws during resorption is the release of hydrogen gas into the bone and adjacent soft tissues. The gas can lead to alterations in the adjacent tissues that may be misinterpreted as soft tissue infection or osteomyelitis. In the long term, the gas may possibly also impair bone growth if it enters the growth plate. Currently, little is known about the appearance of bone and soft tissue alterations due to screw resorption on magnetic resonance imaging (MRI) in children [21,22,23]. It is known that magnesium-based alloy screws cause significantly less artifacts on MRI than titanium screws and thus facilitate radiological follow-up [21,22,24].

The purpose of this single-center prospective study was to evaluate the screw resorption-induced alterations visible on MRI during the most active phase of resorption, i.e., during the first four months after implantation of the screws, whether gas can be detected in the growth plate, and to familiarize radiologists and clinicians with these findings in order to avoid inadvertently misdiagnosing a soft tissue infection or osteomyelitis [25].

## 2. Materials and Methods

### 2.1. Patients

A prospective study was conducted at a single pediatric hospital over a 16-month period between November 2020 and February 2022. All patients included in the study sustained dislocated fractures of the elbow or tibial tuberosity. Patients and/or their parents could voluntarily choose between treatment with conventional titanium screws and resorbable magnesium-based alloy screws.

Those who opted for magnesium screw treatment were invited to participate in this prospective radiological study. Ethics approval was obtained from the local ethics committee. Informed consent was obtained from the parents or patients (if 14 years of age and older). The ethics committee allowed a maximum of two MR examinations per patient as part of the radiological study and no MRI in patients younger than 7 years. Open fractures were not treated with magnesium screws. Demographic data, date of surgery, and clinical data to exclude signs of infection were obtained from the institutional patient database.

### 2.2. Surgical Procedure and Screw

The surgeries were performed by two pediatric trauma surgeons with 20 years (T. K.) and 13 years (C. M.) of experience, respectively. Bioabsorbable magnesium-based compression screws alloyed with Yttrium (MAGNEZIX ^®^ CBS or CS, Syntellix AG, Hannover, Germany) were used to fix the fractures. The diameter of all solid CBS screws was 3.5 mm and that of all hollow CS screws was 4.8 mm. Screw lengths were chosen according to bone size. The surgeons decided which type of screw to use. The alignment of the fracture fragments and drilling of the screw hole were performed with conventional metal tools.

### 2.3. MRI Acquisition

The timing of MR examinations was based on surgical consultations as part of the routine fracture follow-up. MR examinations were performed no earlier than one month after surgery to minimize interference with normal postoperative changes.

All examinations were performed on the same 1.5T scanner (Siemens Avanto fit, Siemens Healthineers, Erlangen, Germany) using a 4-channel flex coil. Sequences and parameters were adapted from a standard clinical routine elbow protocol. A T2-weighted true fast imaging with a steady-state free precession (TRUFI) 3D sequence with water excitation (we) fat suppression, performed in a transverse plane, aligned with the humerus/tibia, was additionally performed to improve gas detection due to the high spatial resolution of the sequence and due to gas causing blooming artifacts on the standard gradient echo technique. Detailed scan parameters are shown in Table 1.

The elbows were placed along the side of the patient, keeping the region of interest in the isocenter of the scanner. All elbows were in a flexed position, with some still in a cast. When the knee was examined, the knee was also placed in the isocenter of the scanner. No sedation was used. The sequences were oriented along the long axis of the humerus or tibia. No metal artifact reduction was performed. Radiological image files were stored in the hospital’s picture archiving and communication system (PACS, Dedalus DeepUnity Diagnost 1.1.0.1, Germany).

### 2.4. MRI Assessment

All MRI examinations were individually evaluated by two pediatric radiologists with an overall experience of 12 years (S. W.) and 7 years (S. M.), respectively. The examinations were assessed independently, blinded, and in a randomized order.

**Figure 1:**
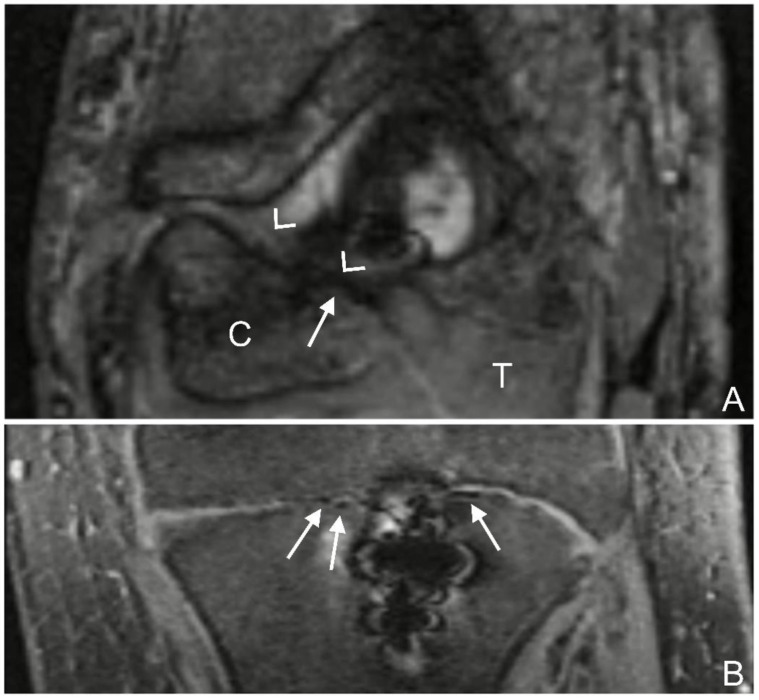
(**A**): T2 trufi 3d we (true fast imaging with steady-state free precession three-dimensional water excitation) coronal image of the left elbow of a 17-year-old boy with a lateral humeral condyle fracture. Gas locules within the growth plate of the humeral capitellum are seen as signal voids (arrow). The humeral capitellum ossification center (C), growth plate (arrow heads), and cartilaginous trochlea (T). (**B**): T2 trufi 3d we coronal image of the tibia of a 12-year-old boy with a tibial tuberosity fracture showing hypointense gas locules (arrows) within the tibial growth plate.

Gas: The bubbly or grape-shaped gas components showed a signal void on all sequences [26,27,28]. The following gas localizations were distinguished: intraosseous adjacent to the screw, intraosseous not in contact with the screw, in the epiphyseal growth plate (if still unfused) (Figure 1A,B), intra-articular, in the soft tissue adjacent to the screw head, and in the soft tissue not in contact with the screw head. The apophyseal growth plates of the medial and lateral epicondyles were not evaluated because they do not contribute to longitudinal growth and are not part of the elbow joint articulation.

**Figure 2:**
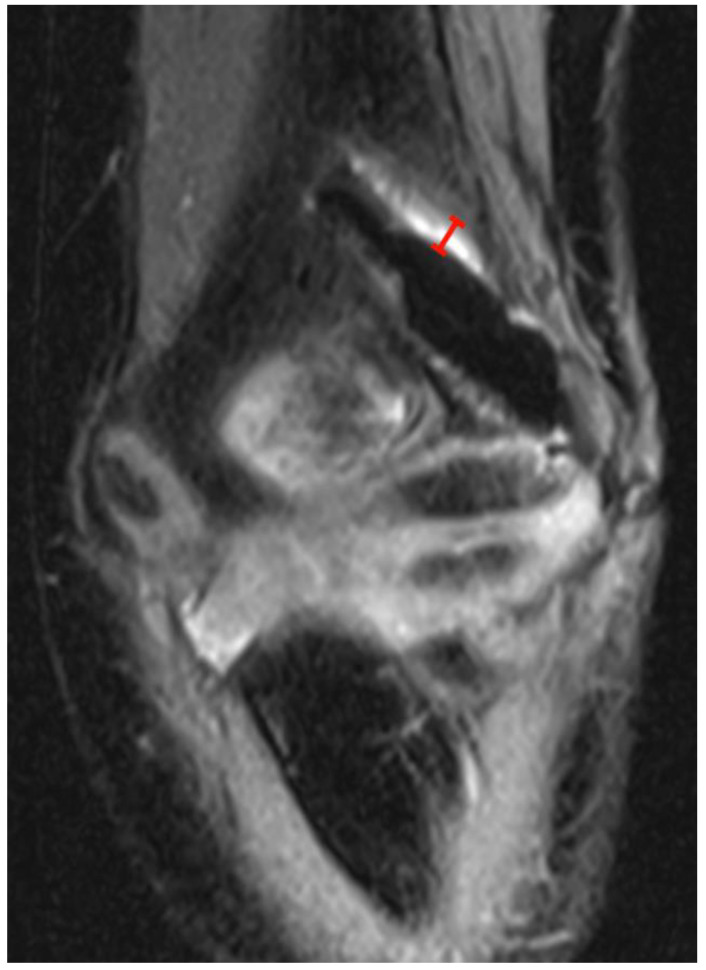
PD tse fs (proton density turbo spin echo fat-saturated) coronal image of the left elbow of a 7-year-old boy with a lateral humeral condyle fracture. The width of the hyperintense layer adjacent to the magnesium screw has been measured (red). Note the irregularity of the hypointense screw caused by gas locules along the screw.

Osteolysis: Similar to previously published studies with titanium screws, a well-defined, hyperintense, linear signal in fluid-sensitive sequences directly adjacent to the screw shaft was interpreted as fluid collection/osteolysis [29]. Its maximum short-axis width was measured (Figure 2).

**Figure 3:**
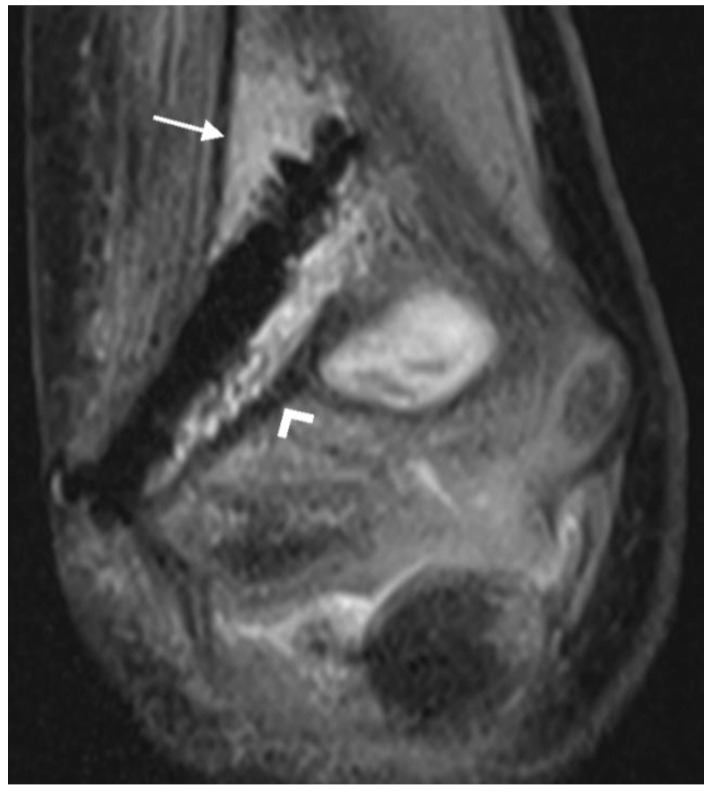
PD tse fs (proton density turbo spin echo fat-saturated) coronal image of the left elbow of a 7-year-old boy with a lateral humeral condyle fracture. Bone marrow edema is seen as a hyperintense signal in the bone marrow (arrow). Hypointense gas locules are also visible along the screw. A second, smaller magnesium screw is visible in the distal part of the humerus (arrowhead).

Bone marrow edema: Bone marrow edema was defined as a hyperintense signal on fluid-sensitive sequences and an intermediate or low T1 signal compared to normal bone marrow [21,30]. A distinction was made between whether the edema was located immediately adjacent to the screw or had no contact with the screw (Figure 3).

**Figure 4:**
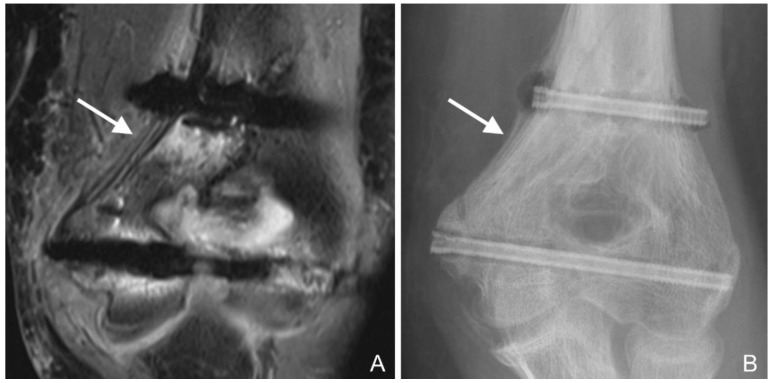
(**A**): PD tse fs (proton density turbo spin echo fat-saturated) coronal image of the left elbow of a 14-year-old boy with an intercondylar humeral fracture. Hypointense layers of ossified periosteal reaction alternate with hyperintense fluid (arrow). Hypointense gas locules along both screws and hyperintense bone marrow edema are also seen. (**B**): Corresponding radiograph. The periosteal reaction is particularly evident near the proximal screw (arrow). The gas locules along the proximal screw and at the screw head are also visible.

Periosteal reaction: A line of low signal intensity separated from the underlying cortex by a layer of high signal intensity fluid was interpreted as ossified periosteum [31,32] (Figure 4A,B).

**Figure 5:**
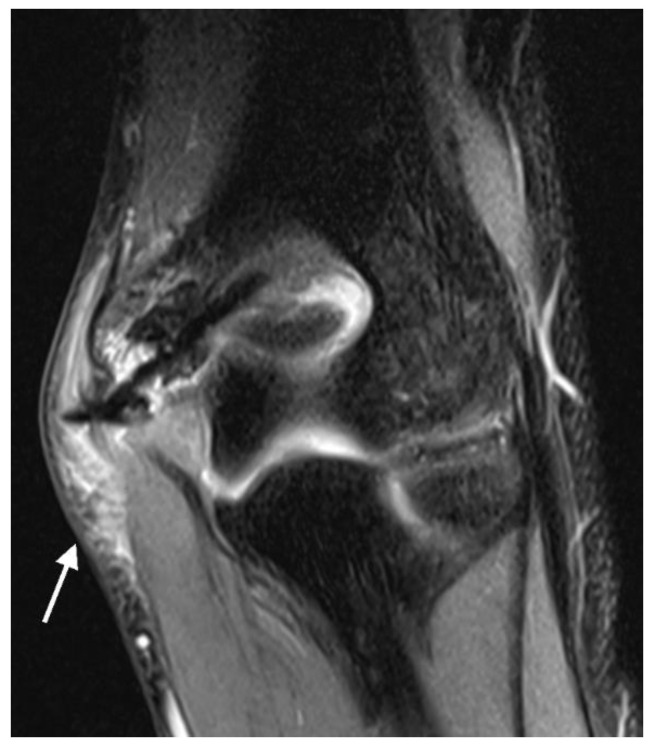
PD tse fs (proton density turbo spin echo fat-saturated) coronal image of the left elbow of a 10-year-old boy with a lateral condyle fracture showing hyperintense soft tissue edema (arrow).

Soft tissue edema: Soft tissue edema was defined as a reticular, hyperintense signal on fluid-sensitive sequences and a hypointense signal on T1-weighted sequences in the subcutaneous fat and superficial fascia [32,33,34]. Two localizations were distinguished: on the side of the screw head (i.e., where the screw is in direct contact with the soft tissues) and on the contralateral side (Figure 5).

**Figure 6:**
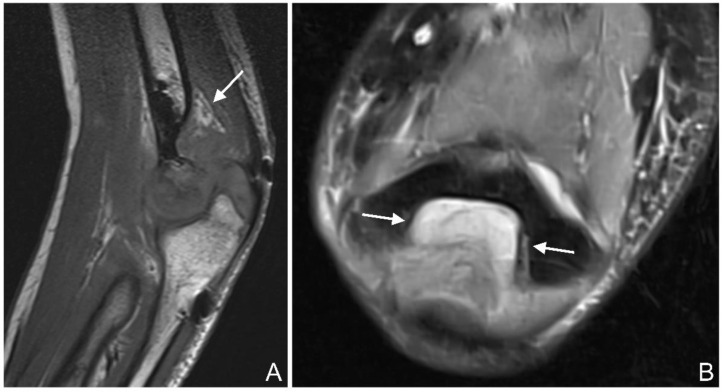
(**A**): T1 se (spin echo) sagittal image of the left elbow of a 17-year-old boy with a lateral humeral condyle fracture (arrow). The posterior fat pad is raised from the olecranon fossa, suggesting a joint effusion. (**B**): PD tse fs (proton density turbo spin echo fat-saturated) transverse image of the left elbow of a 16-year-old girl with a coronoid process fracture. Hyperintense joint effusion (arrow) is seen in the olecranon fossa.

Joint effusion: Joint effusion was defined as increased intra-articular fluid, with the distinction between a normal amount of joint fluid and a pathological effusion being subjective, or if the anterior or posterior fat pads were raised in the case of elbow fractures [35] (Figure 6A,B).

**Figure 7:**
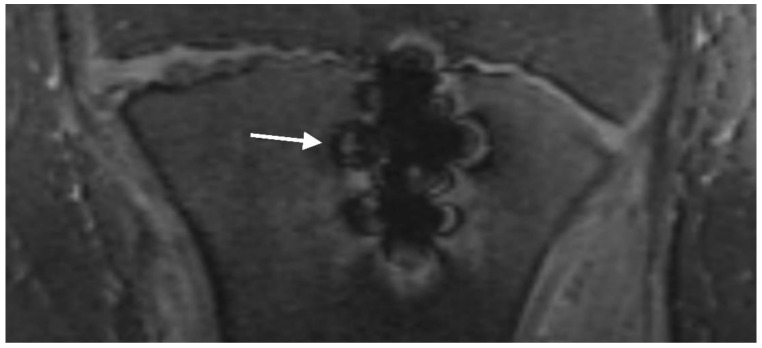
T2 trufi 3d we (true fast imaging with steady-state free precession three-dimensional water excitation) coronal image of the tibia of a 12-year-old boy with a tibial tuberosity fracture showing curvilinear hyperintense signals alternating with bands of signal loss (pile-up artifact) (arrow).

Metal-induced artifacts: MR images were evaluated for the presence of typical metal-induced artifacts generated by the screws (signal pile-up, geometric distortion, failure of fat suppression). Signal pile-up is a curvilinear hyperintense signal that typically follows the contour of the screw and alternates with bands of signal loss, which is well seen in gradient-echo sequences (Figure 7). Geometric distortion causes warping of the normal anatomy adjacent to the screw [36].

**Figure 8:**
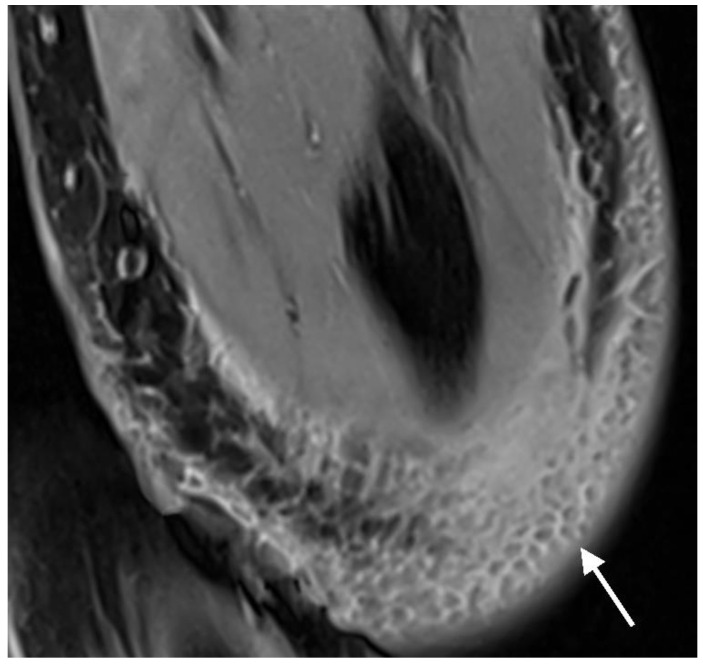
PD tse fs (proton density turbo spin echo fat-saturated) transverse image of the left elbow of a 14-year-old boy with an intercondylar humerus fracture. DIXON fat suppression, visible in the hypointense subcutaneous fat lobules surrounded by hyperintense soft tissue edema (arrow), is unaffected by the magnesium screw and gas (both not visible on this slice).

Signal pile-up and geometric distortion artifacts were assessed using a 4-point Likert scale: no artifacts, minimal artifacts, artifacts impairing diagnostic validity, and no interpretation of surrounding tissue possible. The 4-point Likert scale for fat suppression consisted of the following items: fat suppression not impaired, slightly impaired, heavily impaired but suppressed areas still present, and no fat suppression at all (Figure 8).

If two screws were placed, then of all the parameters described above, the more pronounced finding (e.g., the wider of the two osteolyses) was included in the evaluation.

### 2.5. Statistical Analysis

Given the descriptive nature of this study, and the small number of replicates per patient, we treated each MRI examination as an independent observation. Whenever at least one reader scored a feature of interest to be present, it was recorded as such. Inter-reader agreement was expressed as the number of features for which both readers agreed/the total number of features (n/N), and additionally by Cohen’s kappa for categorical variables. For the only continuous variable of interest (osteolysis), we report the intraclass correlation coefficient (ICC) instead. Whenever the calculation of Cohen’s kappa was not possible (due to perfect agreement), or compromised (due to empty rows/columns in the confusion matrix), we had to fall-back on only reporting agreement in terms of n/N. All statistics and graphs were generated using the ‘pastecs’, ‘psych’, and ‘ggplot’ packages in R version 4.2.1 [37,38,39,40].

## 3. Results

In total, 30 MR examinations from seventeen pediatric patients (six girls, eleven boys) were available for this study. Table 2 summarizes the different fracture types and the respective age of the patients. In total, thirteen patients had two MR examinations each, and four patients had only one MR examination each. The four patients who underwent only one MR examination refused a second examination after the first one. As none of the patients showed clinical signs of infection, no patient had to be excluded. The mean age at the time of surgery was 12.45 years (range 7.30–17.40 years). The median number of days between the fracture and surgery was 1 day (range 0–58 days). The mean time between the surgery and MR examination was 54 days (range 23–102 days).

In the following, percentages have been rounded to the nearest whole number.

**Figure 9:**
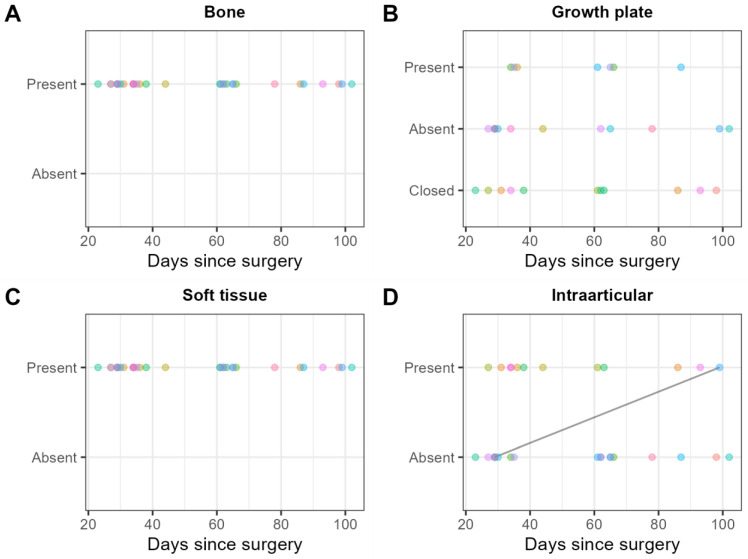
Consensus scores of the two readers on 30 MRI examinations for the presence/absence of gas locules (**A**) in the bone, (**B**) the growth plate, (**C**) the soft tissue, and (**D**) intraarticular. Each patient is represented by one color. Scores obtained on the same patient are shown in the same color. Solid black lines indicate within-patient changes over time.

Gas locules in the bone adjacent to the screw were found in thirty examinations (100%), and without contact to the screw in seven (30%) examinations (Figure 9A). The growth plate was still visible in 19 (63%) examinations. In seven (37%) out of these nineteen examinations, gas locules were found within the growth plate (Figure 9B). Gas locules were found in the soft tissues adjacent to the screw head in 28 (93%) examinations, and not in contact with the screw head in 30 (100%) examinations (Figure 9C). Intra-articular gas locules were found in 12 (40%) examinations (Figure 9D).

Osteolysis adjacent to the screw thread was present in 26 (87%) examinations, with a mean width of 1.5 mm (range 0.60–3.40 mm).

**Figure 10:**
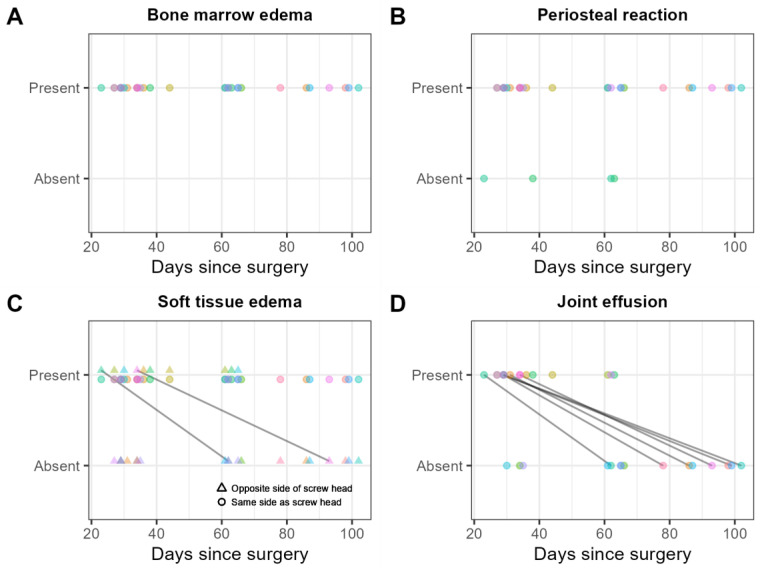
Consensus scores of the two readers on 30 MRI examinations for the presence/absence of (**A**) bone marrow edema in general, that is, whether adjacent to the screw or not in continuity with the edema adjacent to the screw; (**B**) periosteal reaction; (**C**) soft tissue edema in general, that is, whether on the same or the contralateral side of the screw head; and (**D**) joint effusion. Scores obtained from the same patient are shown in the same color. Solid black lines indicate within-patient changes over time.

Bone marrow edema adjacent to the screw was found in thirty (100%) examinations, and not in continuity with the edema adjacent to the screw in three (10%) examinations (Figure 10A).

A periosteal reaction was found in 26 (87%) examinations (Figure 10B).

Soft tissue edema was found on the same side as the screw head in 30 (100%) examinations, and on the contralateral side of the screw head in 10 (33%) examinations (Figure 10C).

Joint effusion was found in 15 (50%) examinations (Figure 10D).

Pile-up artifacts were found in thirty (100%) examinations, of which twenty-six (87%) were minimal, and four (13%) were impairing diagnostic validity. Geometric distortion artifacts were found in zero (0%) examinations.

Chemically selective saturation (CHESS) fat suppression was not impaired in twenty-nine (97%) examinations and was slightly impaired in one (3%) examination. STIR fat suppression was impaired in zero (0%) examinations. The DIXON fat suppression technique was not impaired in twenty-six (87%) examinations and was slightly impaired in four (13%) examinations. Water excitation fat suppression was not impaired in twenty-one (70%) examinations and slightly impaired in nine (30%) examinations.

Inter-reader agreement for gas locules (categorical): Cohen’s kappa was moderate for gas locules in the growth plate (0.744), and intra-articular (0.545) and perfect for gas locules in the bone and in the soft tissues [41]. An overview of the inter-reader agreement for the different variables of interest is given in Table 3.

## 4. Discussion

In this prospective, descriptive study, we have shown the findings typically seen on MRI in the early active stages of resorption of magnesium-based alloy bioabsorbable screws in a pediatric population. We believe that resorbable screws will be increasingly used in pediatric trauma in the future. This young population of patients may require additional imaging, for example, if they have persistent pain or a limited range of motion in the affected joint, and therefore will be examined by MRI due to its lack of radiation. Radiologists and clinicians should thus be familiar with the findings typically seen on MRI in patients with magnesium screws to avoid misdiagnosing pathologies (e.g., osteosynthesis material infection, osteomyelitis, soft tissue infection) where there are none, which may ultimately lead to unnecessary revision surgery and intervention.

In the growing skeleton, the osteosynthesis material is removed in a second surgery after the fracture has healed to avoid interference with growth. Resorbable magnesium screws eliminate the need for a second surgery. Resorption of the screw begins as soon as it comes into contact with chloride ions of the body fluids, which leads to the release of hydrogen gas. To ensure that resorption is not too rapid and excessive, magnesium is alloyed with rare-earth elements, such as yttrium (as in our case) or gadolinium [22,42,43,44,45,46,47,48,49]. Due to the novelty of these screws, little is known about their MR imaging features in children [22,24].

It has been shown that the radiographic findings of screw resorption may resemble those of osteomyelitis or soft tissue infection [25].

Gas: It is known from radiographs in humans, μCT scans in animals, and a recent MR study in adults that hydrogen gas formation is particularly pronounced in the first few weeks and months and then decreases [21,25,49,50,51]. The gas in both the bone and soft tissue is very reminiscent of osteosynthesis material infection, osteomyelitis, or soft tissue infection. In this study, we have shown that bone and soft tissue gas is a normal finding, found in 100% of the examinations.

Growth plate/growth disturbance: Gas within the growth plate was detected in 37% of the MR examinations with unfused growth plates. This is of particular concern in children, as it could potentially lead to growth disturbances. Kraus et al. [49] showed in an in vivo animal study that bone growth was diminished by the resorption of magnesium pin implants (different implants from our study) and the release of hydrogen gas. However, in contrast to our study, the drill hole and subsequently the magnesium implant were placed directly through the growth plate. In this approach, the growth plate may be damaged by the drill and the heat generated during the drilling process, and by the hydrogen gas released during resorption. The implant itself may also impede growth. In our study, the screws were not inserted through the epiphyseal growth plates. In the fractures of the medial humeral epicondyle and the tibial tuberosity, the screws were inserted through the apophyseal growth plates. This was not a problem as there was no significant longitudinal growth emanating from the apophyseal growth plates. In the lateral humeral condyle fractures, the screws were inserted lateral to the epiphyseal growth plate or through the most lateral portion of the growth plate. Premature closure of the epiphyseal growth plates can lead to growth disturbance [52,53,54]. Future long-term studies will have to show whether growth disturbances are more common with the use of magnesium screws.

Osteolysis: New bone is formed adjacent to the screw, but as shown in an animal study, lacunae are also formed [55,56]. These may fill with fluid and correspond to the linear fluid-equivalent hyperintensity along the screw thread seen in 87% of our MR examinations, as has also been shown in adults [21].

Bone marrow edema: In our study, bone marrow edema was seen in 100% of the MR exams. The exact cause of bone marrow edema is not yet clear, but it may be a result of screw resorption. Hydrogen released during screw resorption may react with chloride ions in body fluids and thereby increase the pH value around the screw. The local increase in pH can result in a change of proton content, which in turn affects the MR signal [21,57]. This is in line with the fact that in almost all MR examinations in our study, the bone marrow edema was adjacent to the screw, and only in a few cases was it distant from the screw. However, it is also possible that local increases in magnesium ions lead to a change in osmotic pressure or to changes in the capillary wall, resulting in a fluid shift [21]. A histologic examination of rabbits with cruciate ligament reconstruction using magnesium screws showed no inflammation or necrosis [50]. It may also be (at least in part) post-traumatic edema. Sonnow et al. [21] stated that in their study of 17 adults, bone marrow edema was observed a few weeks after surgery and later resolved, without defining the time of resolution more precisely.

Soft tissue edema: Soft tissue edema adjacent to the screw head was visible in all examinations (100%) and in almost one-third (30%) on the contralateral side. We suspect the same causes as for the bone marrow edema.

Periosteal reaction: A periosteal reaction is very common in children during fracture healing and is not a sign of infection or a finding caused by screw resorption [58]. In our population, a periosteal reaction was seen in 87% of examinations.

Artifacts: The magnetic properties of human tissue and metal screws differ significantly, resulting in local inhomogeneities of the magnetic field [36]. The mass magnetic susceptibility of magnesium is lower than that of titanium, resulting in significantly fewer artifacts with magnesium screws [22,24,59]. 

Signal loss is limited to the screw itself and the screw edges are relatively sharply defined [21]. In our study, only the TRUFI sequence showed pile-up artifacts caused by the screw in 100% of the examinations. No geometric distortion artifacts were found. None of the four fat suppression techniques used were significantly affected by the screw. Of the four fat suppression techniques, the TRUFI sequence, using a water excitation technique, was the most prone to artifacts. However, the band-shaped areas without fat suppression were only visible at the edges of the images and almost never at the level of the screws. We attributed these areas to field inhomogeneities at the interfaces (air/skin), flexion of the elbow joint, and the suboptimal isocentric positioning of the elbow. It is known from metal implants that less artifacts occur when a 1.5T scanner is used instead of a 3T scanner, which is why we used a 1.5T scanner for this study. Meanwhile, Sonnow et al. [21] have shown that the artifacts of magnesium screws do not differ significantly between 1.5T and 3T.

The kappa values seemed to be poor for some features, despite quite good n/N values (e.g., pile-up artifacts: kappa = 0.182, while n/N = 24/30). This was due to the presence of empty rows/columns in the confusion matrix. Kappa attempts to correct for how often readers would agree by chance alone, and in order to do so, it must, among other things, calculate the row/column totals of the confusion matrix. If one of these is zero (or has very few observations), kappa’s estimates become very pessimistic. This is a general problem with all interobserver reliability statistics: their interpretation is very subjective, and easily compromised, especially in smaller datasets.

Not all of the findings reported here are specific to alterations associated with magnesium screw resorption. Bone marrow edema, joint effusion, osteolysis, and periosteal reaction may also be seen in fractures that were not treated or were treated with conventional screws [29,60,61,62].

This study has some limitations. Firstly, the number of patients and MR examinations was small, in part because it was difficult to motivate patients and their parents to partake in these optional MR examinations. Secondly, two different types of screws (hollow and solid) were used. Since we did not expect a significant difference in the findings between the screws and there were only very few cases with hollow screws, we did not separate the results by screw type. Thirdly, each MRI exam was considered individually. However, the majority of patients had two examinations, so the data are clustered. Fourthly, susceptibility artifacts may be caused not only by gas locules but also by metal abrasion, as is often seen in the bone and soft tissue adjacent to the surgical access [63]. Therefore, the frequency of gas locules may therefore be overestimated on MRI. Fifthly, we do not know at this point whether gas in the growth plate really leads to growth disturbances. Many of the children treated with magnesium screws still have several years of growth ahead of them. Long-term studies will have to show whether there are more growth disturbances when magnesium screws are used than when conventional screws are used.

## 5. Conclusions

Edema and gas in the bone and soft tissue adjacent to resorbable magnesium screws are normal and should not be misinterpreted as infection. Gas may also be seen in growth plates. Its significance with respect to growth remains to be determined in future studies. With the exception of pile-up artifacts, resorbable magnesium screws do not cause significant artifacts. In patients with magnesium screws, MRI examinations can be performed without the need for special metal artifact reduction sequences. Standard fat suppression techniques are not significantly affected.

## Figures and Tables

**Table 1:** MRI scanning parameters.

Sequence	Repetition Time (ms)	Echo Time (ms)	Flip Angle (°)	Band-Width (Hz/pix)	Slice Thickness (mm)	Slice Spacing (mm)	FOV (mm)	Fat Suppression
T1w SE sag	630	19	90	65	3	3.3	140 × 140	-
T1w SE cor	460	13	90	125	4	4.4	140 × 140	-
PDw TSE fs cor	2100	24	150	170	3	3.3	140 × 140	CHESS/Fat saturation
PDw TSE fs DIXON tra	3000	14	150	170	4	5.2	120 × 120	DIXON
TIRM cor	4340	22	180	130	4	4.8	140 × 140	STIR
T2w TRUFI 3D WE tra	12.72	5.61	28	150	0.5	overlapping	90 × 90	WE

CHESS = chemically selective saturation, cor = coronal, fs = fat saturated, PD = proton density, sag = sagittal, SE = spin echo, STIR = short tau inversion recovery, TIRM = turbo inversion recovery magnitude, tra = transverse, TRUFI = true fast imaging with steady-state free precision, TSE = turbo spin echo, WE = water excitation.

**Table 2:** Descriptive statistics for the six different fracture types in this study.

Fracture	n/N	%	Age_mean_	Age_range_
Medial epicondyle (humerus)	9/17	52.94	13.31	11.42–17.40
Lateral condyle (humerus)	3/17	17.65	8.42	7.30–9.96
Intercondylar (humerus)	1/17	5.88	13.45	-
Complex distal (humerus)	1/17	5.88	12.63	-
Coronoid process (ulna)	1/17	5.88	14.43	-
Tibial tuberosity	2/17	11.76	12.97	11.44–14.50

Both the total count and percentage of all fractures are given. Mean patient age and age range are reported for the date of surgery.

**Table 3:** Inter-reader agreement on the different variables.

Variable	Agreement (n/N)	Agreement Statistic	95%-CI Around Statistic
Gas locules			
In growth plate	25/30	*κ* _Cohen_ = 0.744	0.544–0.943
Intra-articular gas	24/30	*κ* _Cohen_ = 0.545	0.256–0.835
In bone tissue	30/30	-	-
In soft tissue	30/30	-	-
Osteolysis	*	ICC = 0.500	0.176–0.726
Bone marrow edema	29/30	-	-
Periosteal reaction	23/30	*κ* _Cohen_ = 0.400	0.060–0.740
Joint effusion	24/30	*κ* _Cohen_ = 0.583	0.286–0.880
Pile-up artifacts	24/30	*κ* _Cohen_ = 0.182	−0.214–0.577
Geometric distortion	30/30	*-*	-
Fat suppression			
CHESS	29/30	*-*	-
STIR	30/30	*-*	-
DIXON	26/30	κ _Cohen_ = −0.053	−0.133–0.028
Water excitation	24/30	κ _Cohen_ = 0.412	0.071–0.752

Cohen’s kappa is not defined in the case of perfect agreement. CHESS = chemically selective saturation, STIR = short tau inversion recovery. * Note that osteolysis is a continuous variable, and thus the ratio of the number of MRI exams in which the two readers exactly agreed (n) over the total number of MRI images examined (N) is not informative; furthermore, the ICC instead of *κ* _Cohen_ is calculated.

## Data Availability

The datasets used and/or analyzed during the current study are available from the corresponding author on reasonable request.

## Abbreviations

3D	Three-dimensional
CHESS	Chemically selective saturation
FS	Fat-saturated
MRI	Magnetic resonance imaging
PACS	Picture archiving and communication system
PD	Proton density
SE	Spin echo
STIR	Short tau inversion recovery
T	Tesla
TE	Echo time
TIRM	Turbo inversion recovery magnitude
TR	Repetition time
TRUFI	True fast imaging with steady-state free precession
TSE	Turbo spin echo
W	Weighted
WE	Water excitation
